# Treatment of plasma cell balanitis associated with male genital lichen sclerosus using abrocitinib

**DOI:** 10.1016/j.jdcr.2024.02.010

**Published:** 2024-02-29

**Authors:** Xiaoyu Xiong, Rongyi Chen, Liuyuan Wang, Nanxuan Huang, Lixia Huang, Chunmei Wang, Wujian Ke

**Affiliations:** a Sexually Transmitted Disease Department, Dermatology Hospital of Southern Medical University, Guangzhou, China; b Southern Medical University, Guangzhou, China

**Keywords:** abrocitinib, Janus kinase inhibitor, male genital lichen sclerosus, plasma cell balanitis

## Introduction

Plasma cell balanitis (PCB) is a rare benign skin disease characterized by a shiny and well-defined reddish patch on the glans penis.^1^ Male genital lichen sclerosus (MGLS) is characterized by atrophic ivory plaques, and some scholars link it to autoimmunity.^2^ Both PCB and MGLS can arise from redundant prepuce and exposure to chronic irritation such as prolonged urine infiltration, making their simultaneous occurrence in a patient possible.^3^ Treatments for PCB include calcineurin inhibitors, steroid ointments, and circumcision.^1^ However, achieving a definitive treatment approach that fully satisfies patients with PCB associated with MGLS remains a complex and unmet clinical need.^3^

In recent years, abrocitinib, a selective Janus kinase-1 inhibitor, has gained recognition for its potential in addressing a wide spectrum of inflammatory and autoimmune conditions.^4^ Previous study has shed light on abrocitinib’s promising role in the treatment of MGLS, offering hope to those afflicted by this challenging dermatologic disorder.^2^

We present a successful case study illustrating the application of abrocitinib in the treatment of a patient with concurrent PCB and MGLS.

## Case report

**Fig 1:**
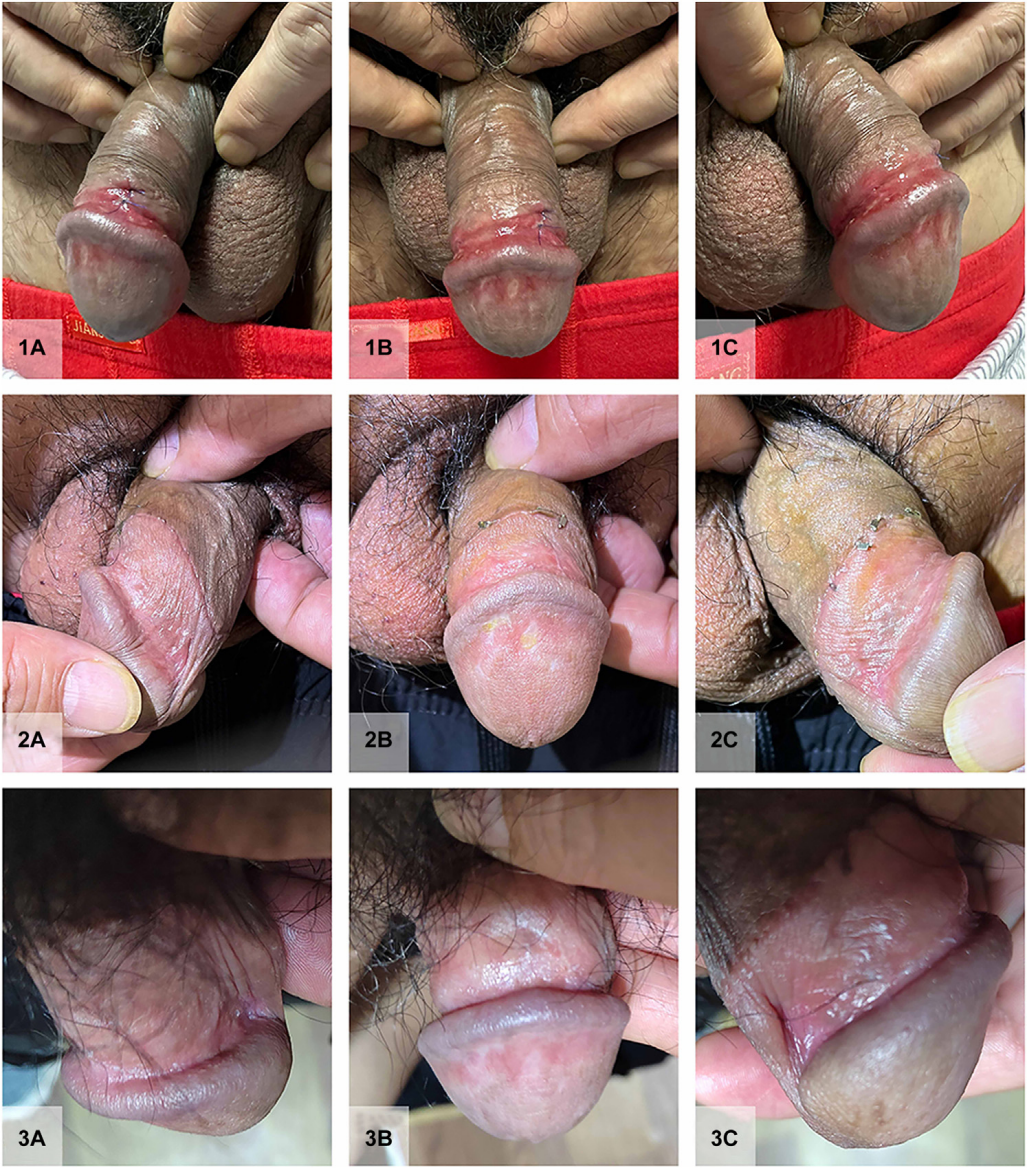
Clinical course of plasma cell balanitis with male genital lichen sclerosus features. **1A-C,** Redundant prepuce, erythematous well-demarcated annular plaques, and erosion on the glans penis. Some of the lesions exhibited white atrophic lines were seen before treatment. **2A-C,** The majority of lesions vanished following 1 month of treatment. **3A-C,** Taken after a 6-month follow-up period, there was no reappearance of the lesions, indicating the sustained efficacy of the treatment.

**Fig 2:**
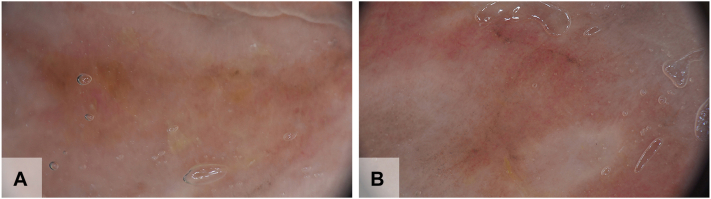
Dermatoscopic characteristics of plasma cell balanitis associated with male genital lichen sclerosus. **A, B,** Dermatoscope of glans before treatment shows spotty, spherical, and atypical vasodilation in a yellow-red background, with some lesions exhibiting erosion and white atrophy.

**Fig 3:**
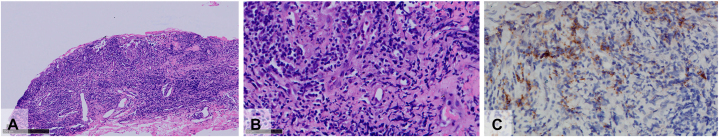
Histopathologic characteristics of plasma cell balanitis associated with male genital lichen sclerosus. In the papillary dermis, there is a diffuse infiltrate of lymphocytes and plasma cells. (**A** and **B,** Hematoxylin-eosin stain; original magnifications: **A**, ×100; **B**, ×400.) CD38 labels a large number of plasma cells. (**C**, Hematoxylin-eosin stain; original magnification: **C,** ×400.)

A 50-year-old man with an 8-month history of genital lesions presented to our sexually transmitted disease department. The patient had been misdiagnosed with genital herpes and bacterial balanitis, resulting in ineffective treatment with antiviral and antibacterial therapies. The patient had a well-controlled history of hepatitis B for over 10 years and no other significant medical history. Physical examination revealed redundant prepuce, erythematous, well-demarcated annular plaques, and erosion on the glans penis (Fig 1, *1A-C*). Some lesions exhibited white atrophic lines. Following admission, comprehensive evaluations, including herpes virus, and syphilis testing, as well as autoimmune antibody screening, were all negative. The dermatoscopic characteristics before treatment (Fig 2, *A*, *B*) consisted of spotty, spherical, and atypical vasodilation with some lesions exhibiting erosion and white atrophy. A large number of plasma cells (Fig 3, *A*, *B*) demonstrated by CD38 positivity (Fig 3, *C*) were present in the histopathologic figures. Therefore, the diagnosis of PCB associated with MGLS is made on the basis of the results of dermatoscopic and histopathologic characteristics.

Initially, we applied 0.03% tacrolimus ointment, 0.05% fluticasone propionate ointment, and 0.1% esacridine solution for 10 days. Unfortunately, the disease progressed, leading to the development of erosion and ulcers. Subsequently, circumcision was performed, but new ulcerations continued to appear. Consequently, we performed a comprehensive analysis, including full blood count, liver, and renal function tests, sexually transmitted disease screening (eg, HIV testing, syphilis serology, and ligase chain reaction for the detection of *Neisseria gonorrhoeae* and *Chlamydia trachomatis*). Before initiating abrocitinib treatment, electrocardiogram and echocardiogram were performed and we conducted additional examinations, assessing the patient’s coagulation function, cardiac function, cardiac function, chest CT, serum tumor markers including carcinoembryonic antigen and alpha fetoprotein, and tuberculosis testing. The results did not indicate a high risk associated with the use of abrocitinib. In addition to the existing treatment, we introduced the Janus kinase-1 inhibitor abrocitinib at a daily oral dose of 100 mg. Remarkably, within 3 days of initiating abrocitinib therapy, the erythematous erosions began to diminish, and by 2 weeks, a significant improvement was observed. The patient did not experience any new ulcers, and complete remission was achieved within the following 1 month (Fig 1, *2A-C*). In the course of a 6-month follow-up, the patient underwent monthly evaluations, including physical examinations (Fig 1, *3A-C*) and an array of tests encompassing liver function, coagulation profiles, complete blood count, lipid indicators, and quantification of hepatitis B DNA. Notably, all results consistently fell within the normal range. Equally significant, the patient remained free from thrombotic events, infections, hepatitis B recurrence, or herpes reactivation throughout this entire follow-up period.

## Discussion

The disease course of PCB and the specific follow-up regimen after treatment remain unclear. Lesions associated with PCB may persist for years before diagnosis, proving to be long-lasting and resistant to treatment. Importantly, failure to respond to topical corticosteroids has been linked to a higher risk of PCB relapse.^5^

The pathogenesis of PCB remains elusive, and it is recognized as a nonspecific inflammatory condition. Many chronic inflammatory skin diseases are associated with the release of proinflammatory cytokines, acting through the intracellular Janus kinase (JAK) or signal transducer and activator of transcription pathway.^4^ IL-6, a crucial inflammatory factor promoting plasma cell proliferation and differentiation, is implicated in this process.^6^ Abrocitinib, through its mechanism of action, may reduce the release of IL-6, thereby mitigating the inflammatory reaction via this signaling pathway and consequently reducing the number of plasma cells in the dermis. Although a definitive therapy for lichen sclerosus remains elusive due to its vague pathogenesis, recent considerations point toward lichen sclerosus as an autoimmune disease with a preference for a Th1 immune response.^7^ Activation of the JAK or signal transducer and activator of transcription pathway has been observed in the pathogenesis of lichen sclerosus. Terlou et al^8^ demonstrated significantly increased expression of proinflammatory cytokines (IFN-γ, CXCR3, CXCL9, CXCL10, CXCL11, CCR5, CCL4, and CCL5) specific to a Th1 IFN-γ-induced immune response.^8^

JAK inhibitors have shown effectiveness in treating atopic dermatitis, alopecia areata, psoriasis, vitiligo, and other diseases.^4^ Studies have highlighted the role of JAK inhibitors in mucosal immunity and tissue repair.^9^

In this case, we successfully treated a patient with PCB associated with MGLS who did not respond to calcineurin inhibitors, steroid ointment, and circumcision. In addition to screening for tumors and chronic infectious diseases before the initiation of therapy (such as hepatitis and tuberculosis), special attention should also be directed toward monitoring for venous thrombosis, anemia, lymphopenia, neutropenia, as well as elevated levels of transaminases, bilirubin, lipids, and creatine phosphokinase.^4^ After starting abrocitinib, his clinical symptoms significantly improved within a few days, with no increased risk of cardiovascular events and infections. This remarkable effect indicates that abrocitinib may present itself as an alternative option for patients who show inadequate response to conventional therapies, such as topical application of steroid ointments, calcineurin inhibitors, and circumcision. However, further research is needed to investigate its long-term efficacy and safety in a larger patient population.

## Conflicts of interest

None disclosed.
